# Assessment of Lymph Node Metastasis in Patients With Gastric Cancer to Identify Those Suitable for Middle Segmental Gastrectomy

**DOI:** 10.1001/jamanetworkopen.2021.1840

**Published:** 2021-03-17

**Authors:** Harbi Khalayleh, Young-Woo Kim, Hong Man Yoon, Keun Won Ryu

**Affiliations:** 1 Center for Gastric Cancer, National Cancer Center, Goyang, Korea; 2 Faculty of Medicine, Hebrew University of Jerusalem, Jerusalem, Israel; 3 The Department of Surgery, Kaplan Medical Center, Rehovot, Israel; 4 National Cancer Center Graduate School of Cancer Science and Policy, Goyang, Korea

## Abstract

**Importance:**

Segmental gastrectomy, a type of function-preserving surgery, is not broadly studied but can improve postoperative function and quality of life among patients with gastric cancer (GC).

**Objective:**

To establish an indication for middle segmental gastrectomy (MSG) as a treatment for middle-body (MB) and high-body (HB) GC.

**Design, Setting, and Participants:**

This cohort study analyzed patients with GC undergoing surgery between January 2000 and December 2015 in the National Cancer Center, Goyang, Korea, a high-volume cancer center with a structured database and accurate long-term follow-up. Inclusion criteria were age 18 to 85 year, histologically proven adenocarcinoma located in the HB or MB, cT1 to cT3 category cancers, curative resection with negative margins performed, and follow-up for at least 3 years. Exclusion criteria were Borrmann type 4 GC, T4 category cancer, neoadjuvant chemotherapy, and a history of other cancers. Data analysis was performed from December 2018 to May 2020.

**Exposures:**

Total or subtotal gastrectomy and LN dissection.

**Main Outcome and Measures:**

The primary outcome was the rate of metastasis at LN stations 2, 4sa, 5, 6, and 11d, which cannot be dissected during MSG.

**Results:**

Among 9952 patients who underwent surgery for GC, 8219 underwent either laparoscopic or open total or subtotal gastrectomy. Seven hundred seventy-three patients (mean [SD] age, 56.21 [12.16] years; 464 men [60.0%]) had GC in the MB or HB of the stomach. Among the 701 patients included in the final analysis after exclusion of the cN2/N3 carcinomas, the mean (SD) age was 56.35 (12.24) years, and 418 (59.6%) were men. The incidence of LN metastasis was 0% at station 5 for cT1-3N0/1M0 cancers, station 4sa for cT1-2N0/1M0 cancers, station 2 for cT1N0/1M0 cancers, station 6 for cT1N1M0 cancers, station 11d for cT1N1M0-cT2N0/1M0 cancers, and station 12a for cT1N0/1M0-T2N1M0 cancers, regardless of size and differentiation. The rates of LN metastasis for cT1N0M0 cancers were 0.3% (1 of 396 LNs) at station 6 and 0.8% (1 of 129 LNs) at station 11d. Tumors 4 cm or smaller were associated with a lower risk of LN metastasis compared with tumors 4.1 cm or larger (odds ratio, 2.10; 95% CI, 1.20-3.67; *P* = .009), and well-differentiated tumors were associated with lower risk of LN metastasis compared with poorly differentiated tumors (odds ratio, 2.88; 95% CI, 1.45-5.73; *P* = .002).

**Conclusions and Relevance:**

These findings suggest that MSG with dissection of stations 1, 3, 4sb, 4d, 7, 8a, 9, 11p, and 12a could be done for HB and MB cT1N0/1M0 gastric cancers 4 cm or smaller and well-differentiated cT2N0/1M0 cancers.

## Key Points

**Question:**

Can metastasis at key lymph node (LN) stations be used to inform surgical management in patients with gastric cancer?

**Findings:**

In this cohort study of 9952 patients who underwent surgery for gastric cancer, the rates of LN metastasis were 0% at LN station 5 for cT1-3N0/1M0 cancers, station 4sa for cT1-2N0/1M0 cancers, station 2 for cT1N0/1M0 cancers, station 6 for cT1N1M0 cancers, station 11d for cT1N1M0-cT2N0/1M0 cancers, and station 12a for cT1N0/1M0-T2N1M0 cancers, regardless of size and differentiation. Well-differentiated tumors were associated with lower rates of LN metastasis vs poorly differentiated tumors, and tumors 4 cm or smaller were also associated with lower rates of LN metastasis vs those 4.1 cm or larger.

**Meaning:**

These findings suggest that middle segmental gastrectomy with dissection of LN stations 1, 3, 4sb, 4d, 7, 8a, 9, 11p, and 12a can be recommended for high-body and middle-body cT1N0/1M0 gastric cancers 4 cm or smaller and well-differentiated cT2N0/1M0 cancers.

## Teaser

This cohort study assesses lymph node (LN) metastasis in patients with gastric cancer to identify those suitable for middle segmental gastrectomy.

## Introduction

Gastric cancer (GC) is the fifth most frequently diagnosed cancer and third leading cause of cancer death worldwide.^1^ Recent developments in screening and diagnostics have elevated the rates of early GC and node-negative cancer in Korea.^2^ Also, the surgical approach has evolved, with the use of minimally invasive surgery.

For the treatment of middle-body (MB) and high-body (HB) GC, distal gastrectomy and total gastrectomy are recognized as standard, safe procedures.^3,4^ However, these procedures are associated with postgastrectomy syndromes that reduce the patient’s quality of life.^5^ Function-preserving procedures, such as pylorus-preserving gastrectomy (PPG), segmental gastrectomy (SG), and proximal gastrectomy (PG), with more limited lymph node (LN) dissection, can improve postoperative quality of life in selected patients.^5,6,7^

We defined middle segmental gastrectomy (MSG) as a small-circumferential gastric resection preserving the cardia and pylorus, excluding PPG.^3,5^ Since the first modification of SG for GC, only a few studies have addressed its safety and advantages.^7,8,9,10,11,12,13,14,15,16^

In MSG, the surgeon does not need to dissect LN station 6, which should be done in PPG.^12,17^ Also, the longer segment of the pyloric antrum can be saved in MSG. Both might contribute to a better functional outcome of MSG.

There is a dearth of data addressing the pattern of LN metastasis according to clinicopathological factors. The risk of LN metastasis is primarily associated with the location of the tumor.^6,18,19^ PPG is usually considered for cancer of the lower body, and PG is suitable for cancer of the upper third of the stomach. However, when the cancer is located in the MB or HB, MSG can be considered, thus minimizing the risk of reflux.^11^ Also, better functional outcome and quality of life are associated with larger remnant stomach volume, which seems to be better achieved in SG than in other function-preserving procedures.^5^ Moreover, knowledge of the expected pattern and rate of LN metastasis in the LN would help surgeons when considering MSG.

Other new methods to estimate LN metastasis, such as sentinel node navigation surgery, which is a combination of nonexposed endoscopic wall-inversion surgery and sentinel node navigation surgery and uses indocyanine green fluorescence imaging and an infrared camera, are still evolving and have noteworthy limitations.^20,21,22,23,24^ Therefore, sentinel node navigation surgery should be validated through clinical trials, and MSG could be one of the procedures based on this concept.^21^ The aim of this cohort study is to determines the rate of LN metastasis for cT1 to cT3 GC in the MB and HB in each nodal station to establish an indication for MSG.

## Methods

This study was approved by the institutional review board of the National Cancer Center of Korea. The institutional review board waived the need for informed consent for this study because the data were deidentified. This study follows the Strengthening the Reporting of Observational Studies in Epidemiology (STROBE) reporting guideline.

To minimize stage migration and to define the criteria for MSG, we calculated the incidence of metastasis independently for each LN station, without reference to the overall pathological stage.^25^ The criteria to decide the eligibility for MSG were location of the tumor in the MB or HB, not GC Borrmann type 4, and incidence of LN metastasis in key stations low enough to ignore the need for subtotal or total gastrectomy or to not lead to an expected benefit when dissecting these stations. An incidence of metastasis at key LN stations (in final pathological analysis) of more than 1.0% should be considered sufficient to argue for dissection.^26^

### Patients

We evaluated the clinical records of 9952 patients who underwent surgery for GC between January 2000 and December 2015 in the Gastric Cancer Center of National Cancer Center, gathered from a prospectively maintained database. Of the 9952 patients, either laparoscopic or open radical total gastrectomy or subtotal gastrectomy was performed in 8219 patients, and of these, 1009 patients had gastric adenocarcinoma located in the MB or HB of the stomach. The inclusion criteria were age 18 to 85 years, histologically proven adenocarcinoma located in the HB or MB, cT1 to cT3 categories, curative R0 (ie, negative margins) resection performed, and postoperative follow-up for at least 3 years. After exclusion of the cases that did not fulfill our criteria, 701 patients who had cT1-3N0/1 adenocarcinomas in the MB or HB were included in the final analysis (eFigure in the Supplement).

### Clinical and Pathological Factors

For staging, the 8th edition of the cTNM classification for GC of the American Joint Committee on Cancer was used, and conversion of the previous version was done. T1a and T1b cancers were grouped together and presented as T1.^27^ The following clinicopathological factors were reviewed: age, sex, location of cancer, longest diameter, histological type, Borrmann type, ulcer (yes or no), the extent of LN dissection, clinical T/N categories, and presence of metastasis in each dissected LN station. The location was defined according to Japanese Gastric Cancer Association classification and based on preoperative esophagogastroduodenoscopy reports.^28^ The clinical T/N categories were determined through preoperative computed tomography. Ulcers were defined as the presence of type 0-III lesions or in combination for early GC or type 2 and 3 lesions for advanced GC. The definition of LN stations was done according to the Japanese Gastric Cancer Association guideline.^3^ We defined the key LN stations (2, 4sa, 5, 6, and 11d) as those that the surgeon will not dissect when performing MSG with D1+ dissection, which is defined as dissection of stations 1, 3, 4sb, 4d, 7, 8a, 9, 11p, and 12a.

### Statistical Analysis

Statistical analyses were performed using SPSS statistical software version 22 (IBM). The association between each clinical factor and the incidence of LN metastasis for each LN station were analyzed by Pearson χ^2^ and Mantel-Haenszel tests. The statistical analysis was done in 2 steps: initial statistical analysis of the 773 cases including cN2/N3 carcinomas and final statistical analysis of 701 cases after exclusion of the cN2/N3 carcinomas. In the univariate analysis, the factors associated with LN metastasis were analyzed using χ^2^ tests, and the factors that were significant in univariate analysis were analyzed by logistic regression analysis for multivariate analysis. Results tested are presented as odds ratio (OR) with 95% CI. Significance was set at 2-tailed *P* < .05. The incidence of LN metastasis at each station was calculated by dividing the number of patients with metastasis at that station by the number of patients in whom that station was dissected, using available pathological reports. Data analysis was performed from December 2018 to May 2020.

## Results

### Initial Statistical Analysis

Among the 773 patients, the mean (SD) age was 56.21 (12.16) years, and 464 (60.0%) were men. A first logistic regression analysis of clinicopathological factors found that factors associated with LN metastasis were tumor size 4.1 cm or larger vs 4 cm or smaller (OR, 2.47; 95% CI, 1.53-3.98; *P* < .001), differentiated vs undifferentiated (OR, 1.63; 95% CI, 1.13-2.37; *P* = .009), cN2/N3 categories vs cN0/cN1 (OR, 6.19; 95% CI, 3.7-10.37; *P* < .001), and cT3 category vs cT1 and cT2 (OR, 13.18; 95% CI, 8.47-20.52; *P* < .001) (Table 1).

**Table 1.** Univariate and Multivariate Regression Analysis of Clinical Risk Factors for Lymph Node Metastasis of 773 Patients With High- or Middle-Body Gastric Cancer

Factor	Patients, No. (N = 773)	Univariate analysis		Multivariate analysis	
		OR (95% CI)	*P* value	OR (95% CI)	*P* value
Age, y					
≤60	465	1.16 (0.83-1.64)	.33	Not included	NA
>60	308	1 [Reference]			
Sex					
Male	464	0.67 (0.47-0.93)	.02	Not included	NA
Female	309	1 [Reference]	<.001		
Clinical tumor mean size, cm					
≤2	341	1 [Reference]	NA	1 [Reference]	NA
2.1-4	350	2.02 (1.4-2.95)	<.001	1.07 (0.68-1.66)	.77
≥4.1	82	3.66 (2.15-6.21)	<.001	1.45 (0.76-2.78)	.25
Binary tumor size category, cm					
≤4	691	1 [Reference]	<.001	NA	NA
≥4.1	82	2.47 (1.53-3.98)			
Tumor differentiation					
Well	110	1 [Reference]	.006	1 [Reference]	NA
Moderate	158	2.12 (1.06-4.23)	.03	1.56 (0.72-3.37)	.25
Poor or signet ring cell carcinoma	505	2.66 (1.44-4.92)	.002	2.43 (1.238-4.79)	.01
Binary differentiation category					
Differentiated^a^	268	1 [Reference]	.009	NA	NA
Undifferentiated	505	1.63 (1.13-2.37)			
Clinical T category					
T1	528	1 [Reference]	<.001	1 [Reference]	<.001
T2	110	4.9 (3.05-7.89)		4.38 (2.54-7.55)	
T3	135	13.18 (8.47-20.52)		10.57 (5.95-18.75)	
Clinical N category					
N0 (0)	554	1 [Reference]	NA	1 [Reference]	NA
N1 (1-2)	147	2.4 (1.6-3.62)	<.001	1.27 (0.78-2.07)	.33
N2 or N3 (>3)	72	6.19 (3.7-10.37)	<.001	1.75 (0.94-3.26)	.08
Ulcer					
No	489	1 [Reference]	<.001	1 [Reference]	.78
Yes	284	3.28 (2.33-4.63)		0.93 (0.58-1.50)	
Esophagogastroduodenoscopy location					
High body	320	1 [Reference]	NA	Not included	NA
Middle body	377	1.15 (0.81-1.65)			
High body and middle body	76	1.38 (0.78-2.45)			

Abbreviations: NA, not applicable; OR, odds ratio.

^a^ Differentiated includes well and moderately differentiated.

The overall rate of LN metastasis for the cN2/3 categories was 55.6% (40 of 72 LNs), and the rates of metastasis in the cN2/3 categories at the key stations were 8.3% (4 of 48 LNs) for station 2, 8.7% (4 of 46 LNs) for station 4sa, 1.7% (1 of 58 LNS) for station 5, 3.4% (2 of 59 LNs) for station 6, and 3.4% (1 of 29 LNs) for station 11d. These rates are higher than the cutoff value (1%) of the predefined criteria for MSG in this study, so we decided to exclude them.

### Second Statistical Analysis After Exclusion of the cN2/N3 Categories

A total of 701 patients were included in the final analysis. The mean (SD) age was 56.35 (12.24) years, and 418 (59.6%) were men. Table 2 shows the background characteristics of the study population.

**Table 2.** Background Characteristics and Preoperative Clinicopathological Findings of the 701 Patients Included in the Second Statistical Analysis

Characteristic	Patients, No. (%) (N = 701)
Age, mean (SD), y	56.35 (12.24)
Sex	
Male	418 (59.6)
Female	283 (40.4)
Extent of LN dissection	
D1+^a^	59 (8.4)
D2^b^	190 (27.1)
D2+^c^	452 (64.5)
Tumor location	
High body	337 (48.1)
Middle body	295 (42.1)
High and middle body	69 (9.8)
Location within wall	
Anterior wall	65 (9.3)
Posterior wall	233 (33.2)
Lesser curvature	238 (34)
Greater curvature	165 (23.5)
Bormann type	
0	566 (80.7)
1	11 (1.6)
2	18 (2.6)
3	106 (15.1)
Differentiation	
Well	105 (15)
Moderate	141 (20.1)
Poor or signet ring cell carcinoma	455 (64.9)
Clinical T category	
T1	516 (73.6)
T2	93 (13.3)
T3	92 (13.1)
Clinical N category	
N0	554 (79)
N1	147 (21)
Size, cm	
≤2	333 (47.5)
2.1-4	304 (43.4)
≥4.1	64 (9.1)
Ulcer presence	
Yes	226 (32.2)
No	475 (67.8)
American Society of Anesthesiologists score	
1	284 (40.5)
2	390 (55.6)
3	27 (3.9)

^a^ D1+ dissection is defined as stations 1 to 7, 8a, 9, and 11p for total gastrectomy and as stations 1, 3, 4sb, 4d, 5, 6, 7, 8a, and 9 for subtotal gastrectomy.

^b^ D2 dissection is defined as stations 1 to 7, 8a, 9, 10, 11p, 11d, and 12a for total gastrectomy and as stations 1, 3, 4sb, 4d, 5, 6, 7, 8a, 9, 11p, and 12a for subtotal gastrectomy.

^c^ D2+ dissection is defined as stations 1 to 7, 8a, 9, 10, 11p, 11d, 12a, and the addition of other stations (eg, 8b, 12b, and 14v) for total gastrectomy and as stations 1, 3, 4sb, 4d, 5, 6, 7, 8a, 9, 11p, 12a and the addition of others (eg, 8b, 12b, and 14v) for subtotal gastrectomy.

### LN Metastasis Rates According to TNM Category

The rates of LN metastasis for the cTNM category at each LN station are presented in Table 3. The incidence of LN metastasis was 0% at station 5 for cT1-3N0/1M0 cancers, station 4sa for cT1-2N0/1M0 cancers, station 2 for cT1N0/1M0 cancers, station 6 for cT1N1M0 cancers, station 11d for cT1N1M0-cT2N0/1M0 cancers, and station 12a for cT1N0/1M0 cancers, regardless of size and differentiation.

**Table 3.** Incidence of LN Metastasis According to Tumor Clinical Classifications

LN station	LN metastases, No./total No. (%)							*P* value
	Total	T1N0M0	T1N1M0	T2N0M0	T2N1M0	T3N0M0	T3N1M0	
All	141/701 (20.1)	50/446 (11.2)	7/70 (10.0)	17/58 (29.3)	15/35 (42.9)	26/50 (52.0)	26/42 (61.9)	<.001
1	30/589 (5.1)	7/377 (1.9)	2/60 (3.3)	5/46 (10.9)	2/28 (7.1)	6/46 (13.0)	8/32 (25.0)	<.001
2	12/374 (3.2)	0/231	0/40	3/32 (9.4)	2/15 (13.3)	3/34 (8.8)	4/22 (18.2)	<.001
3	54/561 (9.6)	20/368 (5.4)	2/55 (3.6)	6/43 (14.0)	7/25 (28.0)	6/40 (15.0)	13/30 (43.3)	<.001
4sa	5/339 (1.5)	0/205	0/41	0/28	0/10	2/34 (5.9)	3/21 (14.3)	<.001
4b	9/545 (1.7)	1/354 (0.3)	2/54 (3.7)	0/42	0/23	3/43 (7.0)	3/29 (10.3)	<.001
4d	18/538 (3.3)	6/351 (1.7)	1/53 (1.9)	2/41 (4.9)	0/25	4/38 (10.5)	5/30 (16.7)	<.001
5	0/600	0/386	0/60	0/47	0/29	0/45	0/33	NR
6	4/612 (0.7)	1/396 (0.3)	0/61	1/46 (2.2)	1/29 (3.4)	0/45	1/35 (2.9)	.05
7	29/604 (4.8)	8/386 (2.1)	2/62 (3.2)	1/47 (2.1)	6/29 (20.7)	5/45 (11.1)	7/35 (20.0)	<.001
8a	10/614 (1.6)	4/394 (1.0)	0/63	0/48	1/29 (3.4)	3/45 (6.7)	2/35 (5.7)	.002
9	14/581 (2.4)	3/376 (0.8)	0/58	0/43	1/28 (3.6)	8/45 (17.8)	2/31 (6.5)	<.001
10	2/178 (1.1)	0/89	0/20	1/22 (4.5)	0/10	1/24 (4.2)	0/13	.20
11p	9/514 (1.8)	3/325 (0.9)	0/55	1/39 (2.6)	0/24	3/38 (7.9)	2/33 (6.1)	.002
11d	4/230 (1.7)	1/129 (0.8)	0/33	0/23	0/9	2/21 (9.5)	1/15 (6.7)	.01
12a	4/443 (0.9)	0/263	0/49	1/41 (2.4)	0/26	1/34 (2.9)	2/30 (6.7)	<.001

Abbreviations: LN, lymph node; NR, not relevant.

The overall rate of LN metastasis at station 2 was 0% for cT1N0/1M0 carcinoma; LN metastasis was found in 3 of 32 cT2N0M0 tumors (9.4%) and 2 of 15 cT2N1M0 tumors (13.3%). These patients had HB poorly differentiated (PD) carcinomas, except for 2 cases, which were well differentiated (WD) and moderately differentiated (MD) and sized 4 and 5 cm according to gastroscopic biopsy; however, in the final pathological analysis, those 2 cases were diagnosed as being PD carcinomas. LN metastasis was diagnosed at station 2 for 3 of 34 cT3N0M0 tumors (8.8%) and 4 of 22 cT3N1M0 tumors (18.2%); and all cases were PD.

The incidence of LN metastasis at station 4sa for T1-2N0/1M0 tumors was 0% irrespective of size, differentiation, and location. For the cT3N0M0 and cT3N1M0 tumors, 2 of 34 (5.9%) and 3 of 21 (14.3) patients, respectively, had a positive node at 4sa station; all these cases were PD tumors.

Overall, 0.7% of patients (4 of 612 patients) diagnosed with LN harbored metastasis at station 6, but this rate was not significantly different between the cTNM categories. One of 396 patients with cT1N0M0 disease (0.3%) had a positive LN at station 6, with a 3-cm MB tumor. One of 46 patients (2.2%) with a cT2N0M0, 1.5-cm, PD carcinoma in the HB had a positive node at station 6, but this patient had stage migration, with T4aN2M0 as the final surgical and pathological classification. The other patient with cT2N1M0 cancer with a positive node at station 6 had a 5-cm carcinoma of the HB with differentiation migration, from preoperative MD to final pathological PD. Finally, 1 cT3N1M0 tumor had a positive node (1 of 35 [2.9%]); this case was a 5-cm PD carcinoma in the esophagogastroduodenoscopy. Excluding these cases with stage migration and size larger than 4 cm, the overall rate of LN metastasis at station 6 for WD and MD cT1-3N0/1M0 tumors is negligible.

The incidence of LN metastasis at the 4d station was 3.3% (18 of 538 cases). The distribution of those cases according to cTNM categories was 6 of 351 cT1N0M0 cancers (1.7%), 1 of 53 cT1N1M0 cancers (1.9%), 2 of 41 cT2N0M0 cancers (4.9%), 4 of 38 cT3N0M0 cancers (10.5%), and 5 of 30 cT3N1M0 cancers (16.7%). It is noteworthy that 14 of 18 patients had tumor in the greater curvature, 2 tumors were in the anterior wall, and 2 tumors were in the lesser curvature, with higher incidence of metastasis in the greater curvature. Fifteen of the 18 LN metastasis–positive patients had carcinoma located in the MB, 3 between the MB and HB, and 3 in the HB, but analysis of the metastatic rate at LN station 4d for MB and HB was not significant. Sixteen of the 18 cases were PD and 2 cases were WD or MD carcinoma.

A total of 4 of 230 patients (1.7%) had a positive node at the 11d station, and the incidence was different between the cTNM categories; 1 of 129 (0.8%) 3.5-cm tumors with a positive node at station 11d was cT1N0M0 classification, and the remaining cases were cT3N0/1M0 categories. All the cases were PD carcinoma located in the HB, except for 1 tumor in the MB.

Throughout the 12a station, the rate of LN metastasis was low (4 of 443 LNs [0.9%]) and differed between TNM categories. One case of 3-cm, PD, cT2N0M0 carcinoma had LN metastasis, and the final pathological analysis showed stage migration to pT3N3M0 disease. Three cases of cT3N0/1M0 disease were all PD, except 1 MD carcinoma.

### LN Metastasis Incidence According to Tumor Size

Logistic regression analysis of the 701 cases shows that tumor metastasis was significantly more common in tumors 4.1 cm or larger (OR, 2.10; 95% CI, 1.20-3.67; *P* = .009) than in smaller tumors. Table 4 shows the logistic regression analysis of the clinicopathological factors associated with LN metastasis incidence.

**Table 4.** Univariate and Multivariate Regression Analysis of Clinical Factors Associated With LN Metastasis of 701 Patients With Cancer Location in the High or Middle Body of the Stomach

Clinical risk factors	Patients, No.	Univariate analysis		Multivariate analysis	
		Odd ratio (95%CI)	*P* value	Odd ratio (95%CI)	*P* value
Age, y					
≤60	416	1.22 (0.83-1.78)	.31	Not included	NA
>60	285	1 [Reference]			
Sex					
Female	283	1 [Reference]	.002	1 [Reference]	.03
Male	418	0.55 (0.38-0.81)		0.61 (0.40-0.94)	
Size, cm					
≤2	333	1 [Reference]	.001	1 [Reference]	NA
2.1-4	304	1.69 (1.13-2.53)	.01	1.11 (0.70-1.77)	.64
≥4.1	64	2.76 (1.51-5.04)	.001	1.64 (0.81-3.29)	.16
Binary size categories, cm					
≤4	637	1 [Reference]	.009	1 [Reference]	.19
≥4.1	64	2.10 (1.20-3.67)		1.53 (0.80-2.92)	
Differentiation					
Well	105	1 [Reference]	.006	1 [Reference]	.05
Moderate	141	2.04 (0.93-4.47)	.07	1.65 (0.70-3.85)	.25
Poor or signet ring cell carcinoma	455	2.88 (1.45-5.73)	.002	2.38 (1.12-5.05)	.02
cT category					
T1	516	1 [Reference]	<.001	1 [Reference]	<.001
T2	93	4.22 (2.540-7.02)		4.09 (2.29-7.29)	
T3	92	10.46 (6.37-17.18)		9.46 (5.12-17.45)	
cN category					
N0 (0)	554	[Reference]	<.001	[Reference]	.23
N1 (1-2)	147	2.40 (1.59-3.62)		1.36 (0.83-2.22)	
Ulcer					
No	475	1 [Reference]	<.001	1 [Reference]	.65
Yes	226	2.46 (1.68-3.59)		0.89 (0.53-1.47)	
Location					
High body	337	0.66 (0.36-1.22)	.19	Not included	NA
Middle body	295	0.70 (0.38-1.28)	.25		
High and middle body	69	1 [Reference]	.41		

Abbreviation: NA, not applicable.

The overall incidence of LN metastasis was observed to be higher for tumors 4.1 cm or larger compared with those 4 cm or smaller (32.8% vs 18.8%). Its noteworthy that the rates of metastasis at key stations were lower in tumors 4 cm or smaller than in tumors 4.1 cm or larger (2.4% vs 9.8% at station 2, 1.3% vs 2.5% at station 4sa, 0% vs 0% at station 5, 0.4% vs 3.7% at station 6, and 1.4% vs 4.3% at station 11d). The rates of LN metastasis for tumors 2 cm or smaller were also low (1.9% at station 2, 1.4% at station 4sa, 0% at station 5, 0.3% at station 6, 0% at station 11d, and 0.5% at station 12a) (eTable in the Supplement).

### LN Metastasis Incidence According to Differentiation

Logistic regression analysis (Table 4) revealed that PD is associated with LN metastasis compared with WD or MD (OR, 2.88; 95% CI, 1.45-5.73; *P* = .002 for univariate analysis; OR, 2.38; 95% CI, 1.12-5.05; *P* = .02 for multivariate analysis). The overall rates of LN metastasis were 9.5% for WD, 17.7% for MD, and 23.3% for PD carcinoma (eTable in the Supplement). The incidence of LN metastasis at stations 4sa, 4sb, 4d, 5, 8a, 9, 11p, 11d, and 12a in WD carcinoma was 0% irrespective of size and TNM category. This incidence was also low at stations 2 and 6 for WD carcinoma, as shown in the eTable in the Supplement.

## Discussion

We attempted to develop an indication for MSG operation based on the risk for LN metastasis to key LN stations. T4 category and Borrmann type 4 GCs should be excluded because of the high risk of LN metastasis. We found that MSG could be feasible for cT1N0-1 GCs 4 cm or smaller in the HB and MB and for WD and MD cT2N0-1 GCs.

We found that tumors 4.1 cm or larger and PD or signet ring cell carcinoma were associated with the rate of LN metastasis compared with tumors 4 cm or smaller and WD or MD histological profile (*P* = .009 and *P* = .002, respectively). The incidence of LN metastasis was 0% at stations 5 for cT1-T3N0/1M0 cancers, 4sa for cT1-T2N0/1M0 cancers, 2 for cT1N0/1M0 cancers, 6 for cT1N1M0 cancers, 11d for cT1N1M0-cT2N0/1M0 cancers, and 12a for cT1N0/1M0 and cT2N1M0 cancers, regardless of tumor size and differentiation.

LN station 6 had metastasis rates of 0.7% for tumors of all sizes and stages and 0.4% for cT1-T3N0/1M0 cancers 4 cm and smaller (eTable in the Supplement). In fact, the 2 cases of LN metastasis at station 6 (1 cT2N0M0 and 1 cT3N1M0) were intraoperatively migrated to category T4a, which make these 2 cases beyond the scope of this study (the surgeon was able to recognize the T4a category visually and to exclude the feasibility for MSG). Thus, MSG seems to be safe with no need for dissection of stations 5 and 6 for carcinoma of the MB and HB with size 4 cm or smaller because the rate of LN metastasis was close to 0% in those stations.

For the cT2N0/1M0 and cT3N0/1M0 cases, the LN metastasis to station 2 was associated with PD carcinoma. Station 2 did not harbor metastasis in WD or MD cancers 4 cm or smaller, which made it possible to consider differentiated T2N0/1M0 carcinomas for MSG, but station 2 of PD cancers still needs to be dissected. Station 4sa was positive only in cT3N0/1M0 cancer and there were differences between the stages, which indicates that T1-T2N0/1M0 carcinoma could be a candidate for MSG candidate from the station 4sa viewpoint.

Only 4 patients had metastasis to station 11d. All of these patients had PD carcinoma, and 3 of them were T3 category, which makes avoidance of dissection of station 11d safe in cT1-T2N0/1M0 cancer.

We noted that station 4d is not a key station but was mainly positive in patients with cT1-T2N0/1M0 tumors located in the greater curvature. Thus, the surgeon can consider saving station 4d in cases with tumor location other than the greater curvature.

Our analysis confirmed that cT1N0M0 and cT1N1M0 carcinomas 4 cm or smaller are eligible for MSG with no need for dissection of the key LN stations. However, MSG could be considered by the surgeon for T1 category cancers when endoscopic submucosal dissection is not indicated. Regarding cT2N0M0 and cT2N1M0 cancers, station 2 had an elevated rate of LN metastasis in PD cancer only, which confirms the need for standard surgery or PG with dissection of this station. However, the cT2N0M0 and cT2N1M0 WD and MD carcinomas 4 cm or smaller had a rate of LN metastasis to key stations close to 0% and can safely undergo MSG. The Figure suggests an algorithm for analyzing the feasibility of MSG in HB and MB cancer.

**Figure.**
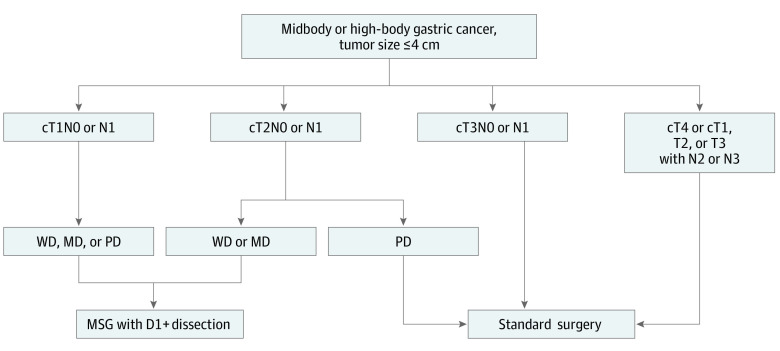
Algorithm of Middle Segmental Gastrectomy (MSG) for Middle-Body and High-Body Gastric Cancer D1+ indicates dissection of stations 1, 3, 4sb, 4d, 7, 8a, 9, 11p, and 12a (station 4d can be reserved if the tumor is not in the greater curvature); MD, moderately differentiated; PD, poorly differentiated; WD, well differentiated.

Our findings are consistent with those of other authors. Furukawa et al^8^ performed SG for MB cancer limited to the mucosal layer (T1) and smaller than 2 cm and reported a low recurrence rate after SG. Furukawa et al^8^ also reported fewer postoperative complications and less cholelithiasis after SG than after subtotal gastrectomy, and they also used dissection of LNs along the resected stomach wall (ie, D0-1 dissection) instead of dissection of stations 1, 3, 4sb, 4d, 7, 8a, 9, 11p, and 12a.

Ohwada et al^9^ described the use of SG in 30 patients with different sized (4 were 1 cm, 14 were 1-2 cm, 11 were 2.1-5 cm, and 1 was ≥5.1 cm) mucosal (23 cases) and submucosal (7 cases) MB cancers and found 100% disease-free survival during 30 months of follow-up. These authors also reported that the dietary volume returned to preoperative levels after 12 months.

Shinohara et al^11^ reported the use of MSG (instead of PG) for HB cancer with favorable survival. The procedure was confirmed to reduce the reflux that is usually associated with PG.

Matsuda et al^15^ have described the use of a new modified D2 dissection (ie, dissection of stations 1, 2, 3, 4sb, 4d, 5, 6, 7, 8a, 8p, 9, 11p, and 12a) during SG for 14 MB or lower-body GCs and found just 2 cases with LN metastasis to stations 7 or 3 in the final pathological analysis. The 14 cases included 4 mucosal, 8 submucosal, 1 muscularis, and 1 subserosal cancers. These findings are consistent with our finding that the rate of LN metastasis in the key stations could be negligible in T1 and differentiated T2 tumors. In contrast to the study of Matsuda et al,^15^ the present study suggests the use of MSG for MB or HB cancers, not lower-body cancers, because we believe that PPG is more feasible for lower-body cancer.

Ishikawa et al^14^ used SG for WD and MD mucosal cancers less than 3 cm in diameter in the middle third of the stomach and found no LN metastasis in 33 SG cases and no recurrence during follow-up (mean, 54.7 months). Iseki et al^16^ used an indication of T1 to T2 cancer, size 5 cm or smaller, and location in the middle third or distal half of the upper third of the stomach (HB) for SG. These authors studied 100 cases and reported a 97% survival rate after 5 years, which supports the oncological safety of SG.

### Strengths and Limitations

A strength of this study is its use of clinical stage to define patient eligibility for MSG and its careful analyses of each preoperative clinical factor. Limitations of this study include its retrospective nature and the possibility of some stage migration (between T2 and T3 tumors). Also, not all patients had detailed LN data available. A prospective clinical trial is now needed to assess MSG feasibility for cT1-T2 tumors, where endoscopic submucosal dissection is not indicated.

## Conclusions

This cohort study found that the rates of LN metastasis to key stations for cT1N0/1M0 tumors and differentiated cT2N0/1M0 tumors 4 cm or smaller located in the MB or HB were low enough to make MSG feasible.

## Notes

Supplement. **eFigure.** Patient Selection Flow Diagram **eTable.** Incidence of LN Metastasis According to Tumor Size and Histological Differentiation
