# Supplementary material for: Assessment of Lymph Node Metastasis in Patients With Gastric Cancer to Identify Those Suitable for Middle Segmental Gastrectomy
Source: JAMA Netw Open. 2021 Mar 17;4(3):e211840. doi: 10.1001/jamanetworkopen.2021.1840 (PMC7970333; doi:10.1001/jamanetworkopen.2021.1840)
Supplement: Supplement. — eFigure. Patient Selection Flow Diagram eTable. Incidence of LN Metastasis According to Tumor Size and Histological Differentiation [file jamanetwopen-e211840-s001.pdf]

Supplemental Online Content

Khalayleh H, Kim YW, Yoon HM, Ryu KW. Assessment of lymph node metastasis in patients with gastric cancer to identify those suitable for middle segmental gastrectomy. *JAMA Netw Open*. 2021;4(3):e211840. doi:10.1001/jamanetworkopen.2021.1840

eFigure. Patient Selection Flow Diagram

eTable. Incidence of LN Metastasis According to Tumor Size and Histological Differentiation

This supplemental material has been provided by the authors to give readers additional information about their work.

© 2021 Khalayleh H et al. *JAMA Network Open.*

eFigure: Patient Selection Flow Diagram

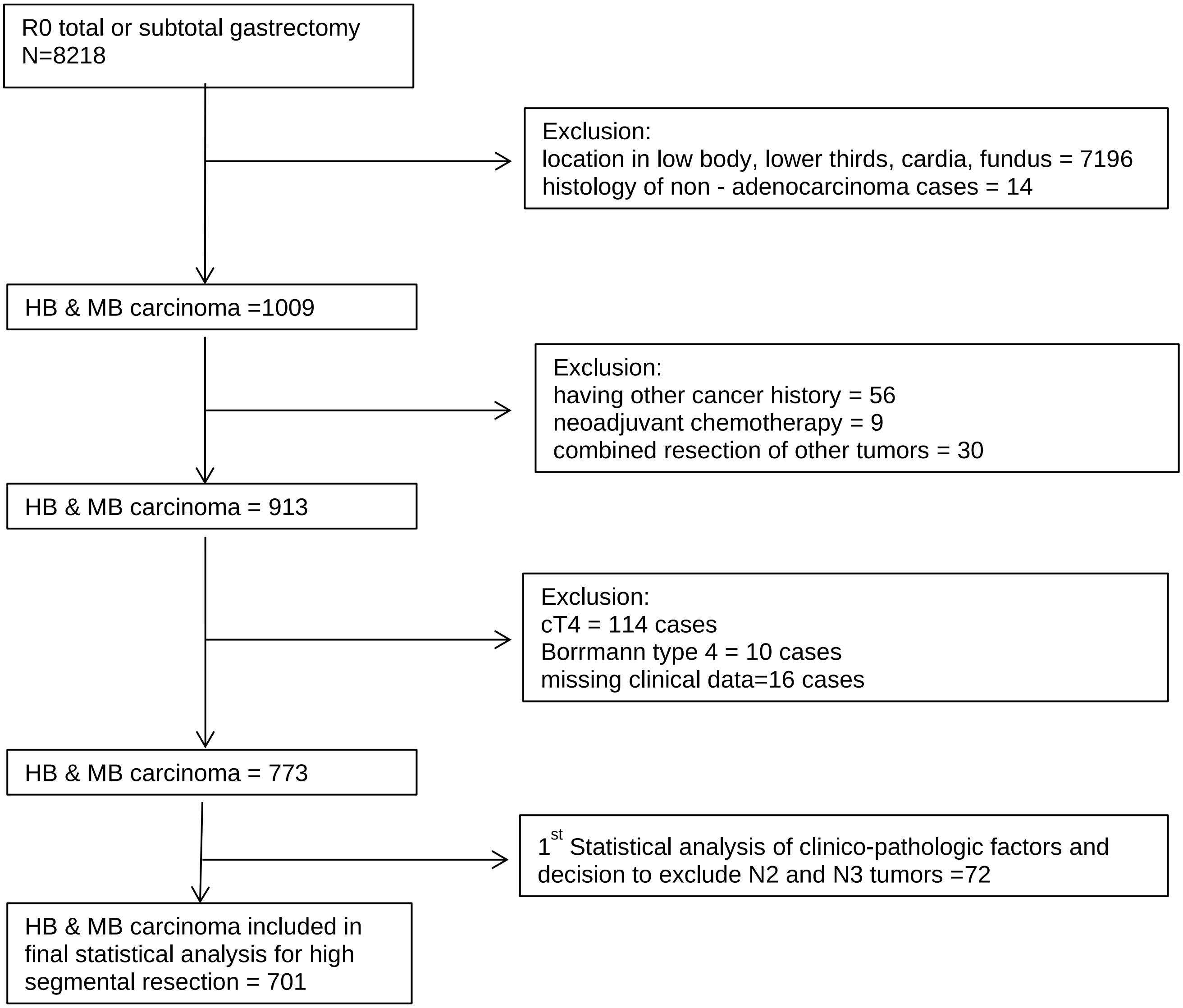

© 2021 Khalayleh H et al. *JAMA Network Open.*

eTable: Incidence of LN Metastasis According to Tumor Size and Histological Differentiation

LN station	Total	≤2 cm (%)	2.1-4 cm (%)	≥4.1 cm (%)	P value	WD (%)	MD (%)	PD (%)	P value
All nodes	141/701 (20.1)	50/333 (15)	70/304 (23)	21/64 (32.8)	0.000	10/105 (9.5)	25/141 (17.7)	106/455 (23.3)	0.001
1	30/589 (5.1)	8/283 (2.8)	16/254 (6.3)	6/52 (11.5)	0.005	2/87 (2.3)	4/112 (3.6)	24/390 (6.2)	0.096
2	12/374 (3.2)	3/158 (1.9)	5/175 (2.9)	4/41 (9.8)	0.035	1/55 (1.8)	3/79 (3.8)	8/240 (3.3)	0.672
3	54/561 (9.6)	18/266 (6.8)	27/242 (11.2)	9/53 (17)	0.012	2/84 (2.4)	12/100 (12)	40/377 (10.6)	0.059
4sa	5/339 (1.5)	2/146 (1.4)	2/153 (1.3)	1/40 (2.5)	0.706	0/49 (0)	0/70 (0)	5/220 (2.3)	0.129
4b	9/545 (1.7)	3/363 (1.1)	6/234 (2.6)	0/48 (0)	0.774	0/73 (0)	1/101 (1)	8/371 (2.2)	0.353
4d	18/538 (3.3)	8/257 (3.1)	8/233 (3.4)	2/48 (4.2)	0.712	1/73 (1.4)	1/101 (1)	16/364 (4.4)	0.08
5	0/600 (0)	0/286 (0)	0/259 (0)	0/55 (0)	NR	0/91 (0)	0/113 (0)	0/396 (0)	NR
6	4/612 (0.7)	1/295 (0.3)	1/263 (0.4)	2/54 (3.7)	0.045	1/94 (1.1)	1/116 (0.9)	2/402 (0.5)	0.496
7	29/604 (4.8)	7/290 (2.4)	16/260 (6.2)	6/54 (11.1)	0.002	4/92 (4.3)	1/114 (0.9)	24/398 (6)	0.175
8a	10/614 (1.6)	4/292 (1.4)	4/267 (1.5)	2/55 (3.6)	0.358	0/95 (0)	2/117 (1.7)	8/402 (2)	0.201
9	14/581 (2.4)	5/282 (1.8)	8/251 (3.2)	1/48 (2.1)	0.487	0/87 (0)	3/114 (2.6)	11/380 (2.9)	0.151
10	2/178 (1.10)	1/77 (1.3)	1/76 (1.3)	0/25 (0)	0.673	1/27 (3.7)	0/32 (0)	1/119 (0.8)	0.324
11p	9/514 (1.8)	2/246 (0.8)	5/221 (2.3)	2/47 (4.3)	0.071	0/66 (0)	1/105 (1)	8/343 (2.3)	0.137
11d	4/230 (1.7)	0/97 (0)	3/110 (2.7)	1/23 (4.3)	0.075	0/28 (0)	0/48 (0)	4/154 (2.6)	0.194
12a	4/443 (0.9)	1/203 (0.5)	2/202 (1)	1/38 (2.6)	0.240	0/61 (0)	1/87 (1.1)	3/295 (1)	0.539

LN: lymph node, WD: well differentiation, MD: moderate differentiation, PD: poor differentiation (include signet ring cells carcinoma), NR: not relevant.

© 2021 Khalayleh H et al. *JAMA Network Open.*
